# 

**DOI:** 10.1192/bjb.2024.92

**Published:** 2025-06

**Authors:** Nyembezi Faith Ndebele

**Affiliations:** Consultant psychiatrist at Hampshire and Isle of Wight Healthcare NHS Foundation Trust, Southampton, UK. Email: faith.ndebele@solent.nhs.uk

**Figure:**
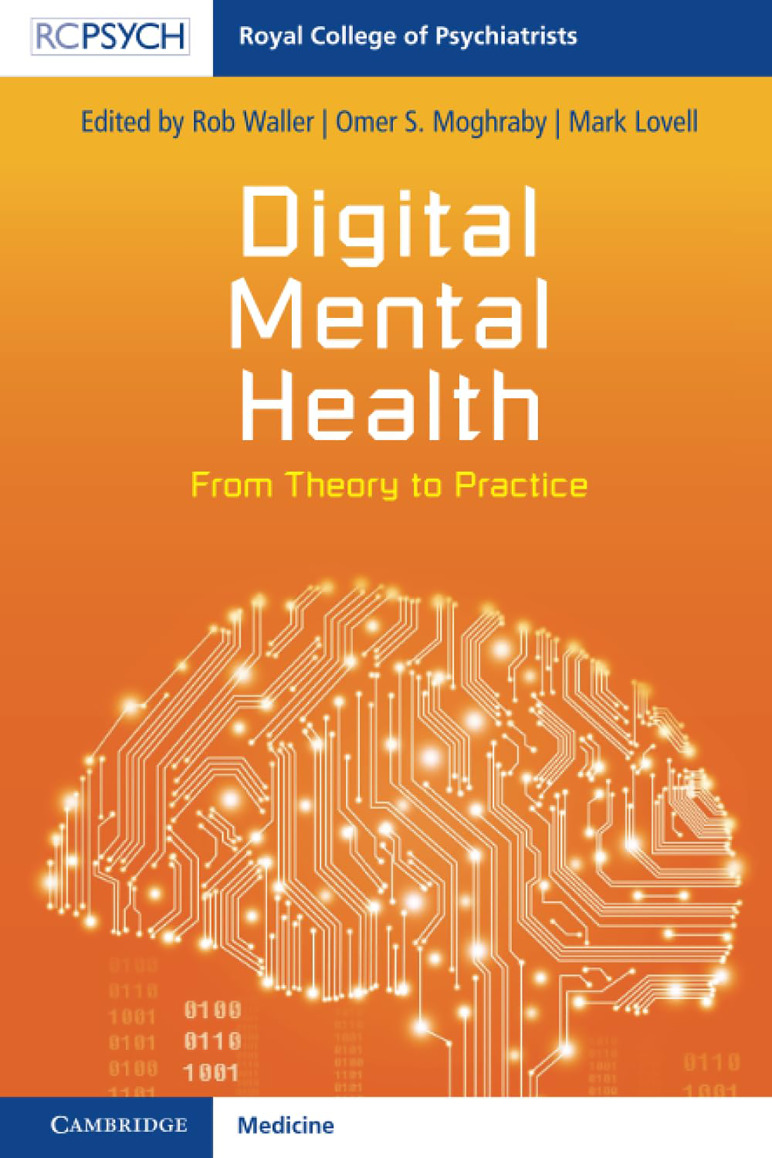

This book is intended for all mental health professionals and aims to give an update on key areas in digital mental health. Its goal is to address what can be reasonably accomplished by digital mental healthcare in the next 10 years.

Written by multiple contributors, it covers a number of themes, beginning with a discussion on the benefits and challenges of information technology (IT) systems in the National Health Service in broad terms before exploring technology-enabled care in more detail and then considering how electronic record systems can be developed to support the safe delivery of care.

The chapter on big data highlights the importance of patient and public involvement and looks at, among other things, advances in technology and computing and how they provide opportunities for analysing data on a large scale. The chapter on artificial intelligence provides a good introduction to the subject and discusses the advantages and dangers of its use in psychiatry.

The importance of the development of digital clinicians is noted and there is a chapter on global telepsychiatry that highlights the rapid digital transformation that has taken place as a result of the COVID-19 pandemic and discusses how psychiatric practice has changed in recent years with more widespread use of video conferencing and hybrid working. The case studies from various countries illustrate the global transformation of psychiatry. Various innovations are discussed, including hybrid psychiatric care, real-time video conferencing and asynchronous telepsychiatry and it is noted that, post-pandemic, online mental health systems will likely be integrated into the way care is delivered.

The book ends with a discussion on how technology can be used well, with useful digital well-being tips included and the reminder that connection with others is important and social interactions are still key.

In the conclusion it is reiterated that both digitisation and digital transformation are required in healthcare and that clinicians should be at the heart of this.

Although there is mention in a number of places in the 10 chapters of the challenges of the digital divide and health inequalities, this is something that could be discussed in more detail. The book was written in the middle of a global pandemic which fundamentally changed the way we interact with technology in psychiatry and the ambition was to look at what the next 10 years will look like. As technology has the potential to change extremely fast this is a difficult task as it can be challenging to predict what these changes might mean in practice. Artificial intelligence, for example, will likely impact and significantly change the way mental healthcare is delivered in the future, so it is ambitious to predict what the future in 10 years might look like.

This is an essential book that gives an excellent introduction to digital mental health and offers a glimpse into what the future could look like. I would recommend it to every psychiatrist and mental health professional.

